# Supplementary material for: Genomics Confirm an Alarming Status of the Genetic Diversity of Belgian Red and Belgian White Red Cattle
Source: Animals (Basel). 2021 Dec 16;11(12):3574. doi: 10.3390/ani11123574 (PMC8697887; doi:10.3390/ani11123574)
Supplement: Supplementary file 1 [file animals-11-03574-s001.zip › Supplementary Material _ Figures S1 S2 S3.pdf]

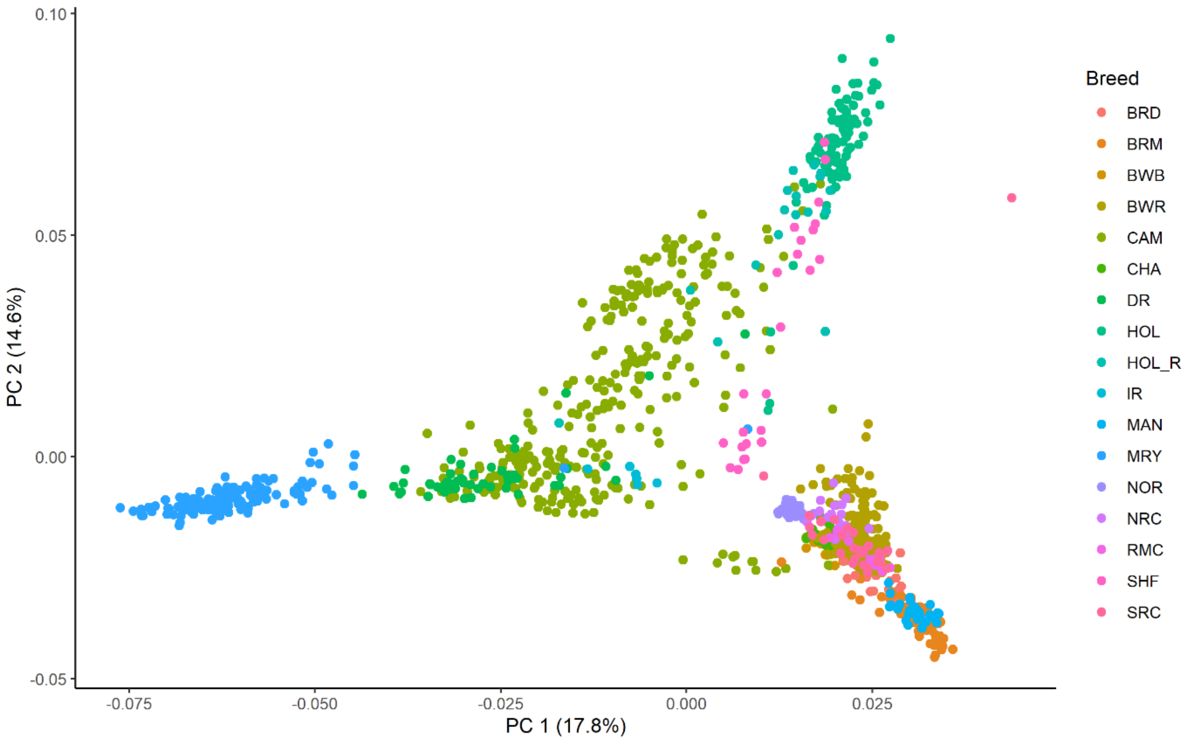

Additional Figure S1. Principal component analyses of Belgian Red (meat type: BRM, and dual purpose: BRD) and Belgian White Red (BWR) compared to the 14 other selected populations. This figure shows first principal component scores on the x-axis and second principal component scores on the y-axis, together they explain 32.4% of the total observed variance. Abbreviations as in Additional Table 1.

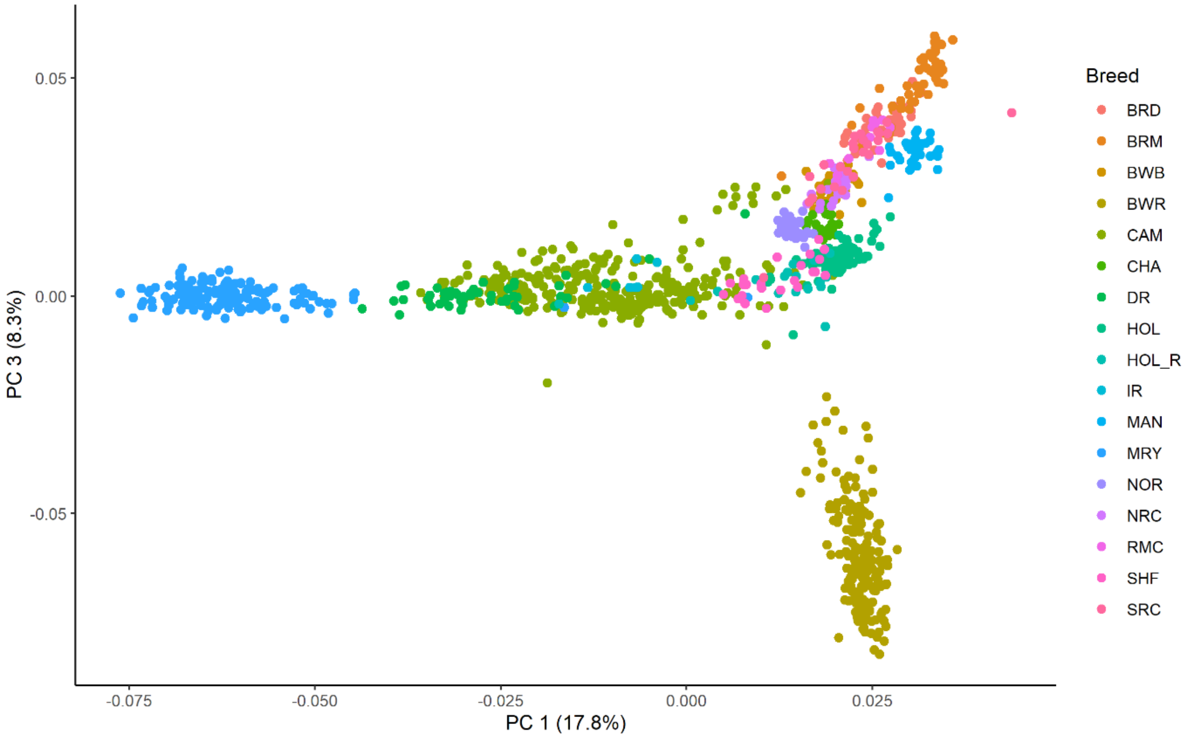

Additional Figure S2. Principal component analyses of Belgian Red (meat type: BRM, and dual purpose: BRD) and Belgian White Red (BWR) compared to the 14 other selected populations. This figure shows first principal component scores on the x-axis and third principal component scores on the y-axis, together they explain 26.1% of the total observed variance. Abbreviations as in Additional Table 1.

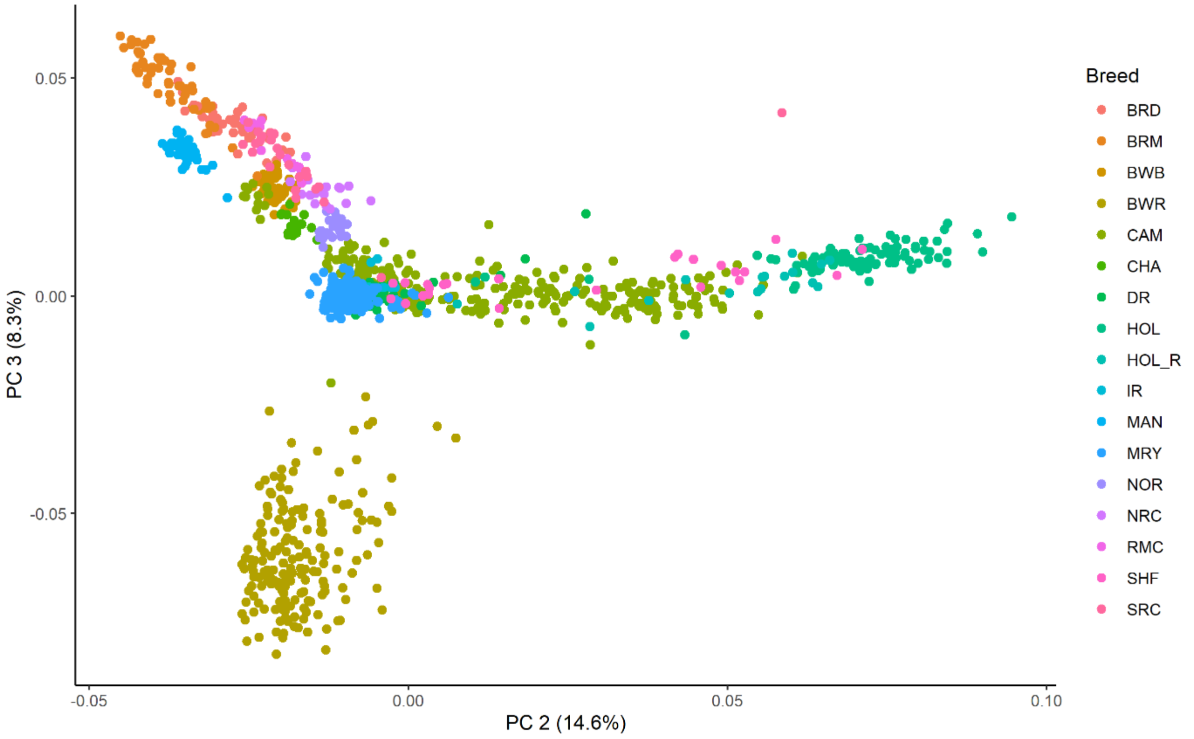

Additional Figure S3. Principal component analyses of Belgian Red (meat type: BRM, and dual purpose: BRD) and Belgian White Red (BWR) compared to the 14 other selected populations. This figure shows second principal component scores on the x-axis and third principal component scores on the y-axis, together they explain 22.9% of the total observed variance. Abbreviations as in Additional Table 1.
