# Supplementary material for: Genomics Confirm an Alarming Status of the Genetic Diversity of Belgian Red and Belgian White Red Cattle
Source: Animals (Basel). 2021 Dec 16;11(12):3574. doi: 10.3390/ani11123574 (PMC8697887; doi:10.3390/ani11123574)
Supplement: Supplementary file 1 [file animals-11-03574-s001.zip › Supplementary Material _ Table S1.pdf]

Additional Table S1. Overview of all available populations in the inter-breed analysis with their used abbreviation, the number of genotyped animals (N), the full breed name and their reference.

Abbrev.	N	Breed	Ref	Abbrev.	N	Breed	Ref
ABO	22	Abondance	(22)	MAL	30	Mallorquina	(23)
ANG	61	Angus	(24)	MAN	34	Maine Anjou (Rouge des Près)	(22)
AUB	22	Aubrac	(22)	MAR	18	Maraichine (Parthenaise)	(22)
BHP	7	Bohus Polled cattle	(20)	MEN	30	Menorquina	(23)
BLO	5	Blonde d'Aquitaine	(25)	MON	30	Montbeliarde	(22)
BPN	18	Bretonne Black Pied	(22)	MRY	150	Meuse-Rhine-Yssel	(9)
BRM	53	Belgian Red (meat type)	this study	NAN	5	Negra Andaluza	(23)
BRD	38	Belgian Red (dual purpose)	this study	NAV	30	Casta Navarra	(23)
BRU	18	French Brown Swiss	(22)	NOR	30	Normande	(22)
BSW	22	Brown Swiss	(25)	NRC	21	Norwegian Red Cattle	(24)
BWB	50	Belgian Blue	(9)	PMT	24	Piedmontese	(24)
BWR	179	East-Flemish White & Red	this study	PRP	72	French Red Pied Lowland	(22)
CAM	286	Campine	(9)	RDB	29	Raço di Biou	(23)
CHA	20	Charolais	(22)	RGU	15	Red Angus	(24)
CHL	26	Charolais (UK pop)	(24)	RMC	13	Ringamåla cattle	(20)
COR	33	Corse	(23)	RMG	24	Romagnola	(24)
CYP	8	Cyprus	(23)	SAL	21	Salers	(22)
DR	51	Deep red	(9)	SAM	11	Sardp-Modicana	(23)
FNC	16	Fjällnära cattle	(20)	SAR	12	Sarda	(23)
GAS	22	Gascon	(22)	SHF	24	Swedish Holstein-Friesian	(20)
GNS	21	Guernsey	(24)	SMC	23	Fjäll cattle	(20)
HFD	31	Hereford	(24)	SPC	12	Swedish Polled cattle	(20)
HOL	93	Holstein	(22) and (24)	SRC	25	Swedish Red cattle	(20)
HOL_R	17	Holstein Red	(9)	SRP	18	(20)	(20)
IR	6	Improved Red	(9)	TAR	17	Tarine	(22)
JER	49	Jersey	(22) and (24)	VAC	9	Väne cattle	(20)
LMS	44	Limousin	(24)	VOS	10	Vosgienne	(22)
MAA	22	Marsimeña	(23)				
